# Root Extracts From *Ononis spinosa* Inhibit IL-8 Release *via* Interactions With Toll-Like Receptor 4 and Lipopolysaccharide

**DOI:** 10.3389/fphar.2020.00889

**Published:** 2020-06-12

**Authors:** Verena Spiegler, Barbara Gierlikowska, Thorsten Saenger, John N. Addotey, Jandirk Sendker, Joachim Jose, Anna K. Kiss, Andreas Hensel

**Affiliations:** ^1^Institute of Pharmaceutical Biology and Phytochemistry, University of Münster, Münster, Germany; ^2^Department of Laboratory Diagnostics and Clinical Immunology of Developmental Age, Medical University of Warsaw, Warsaw, Poland; ^3^Institute of Pharmaceutical and Medicinal Chemistry, University of Münster, Münster, Germany; ^4^Department of Pharmaceutical Chemistry, Faculty of Pharmacy and Pharmaceutical Sciences, Kwame Nkrumah University of Science and Technology, Kumasi, Ghana; ^5^Department of Pharmacognosy and Molecular Basis of Phytotherapy, Medical University of Warsaw, Warsaw, Poland

**Keywords:** *Ononis spinosa*, restharrow root, inflammation, Toll-like receptor-4, clitorienolactone B, CD11b/CD18, CD62L

## Abstract

Extracts from the roots of *Ononis spinosa* L. (restharrow roots) are traditionally used for the treatment of patients with urinary tract infections due to its mild diuretic activity, caused by the inhibition of renal human hyaluronidase-1 by isoflavonoids. Preliminary studies also indicated anti-inflammatory effects. The following study aimed at investigating potential anti-inflammatory effects of restharrow extracts, prepared with solvents of different polarity. A dichloromethane extract (OS1), mainly composed of isoflavonoids and triterpenes as characterized by LC-MS, showed a concentration-dependent (25–100 μg/ml) inhibition of IL-8 and TNF-α release from LPS-stimulated human neutrophils. Significant inhibition was also found for the triterpene α-onocerin and the norneolignan clitorienolactone B, isolated from OS1. Further, OS1 and both compounds significantly decreased the expression of the adhesion molecules CD11b/CD18 and conversely increased the expression of CD62L in LPS-stimulated human neutrophils. This finding corresponds to a reduced inflammatory response by the inhibition of adhesion and migration of immune cells. As all of the observed effects are potentially mediated *via* Toll-like receptor 4 (TLR4) signaling, TLR4 transfected HEK293 cells were incubated with OS1. LPS-induced IL-8 secretion was significantly inhibited in a concentration-dependent manner, confirming TLR4 antagonism. This inhibition, however, was in part caused by an interaction of OS1 with LPS. In addition, also an aqueous extract containing high amounts of isoflavonoid glycosides and saponins from the roots of *O. spinosa* showed anti-inflammatory effects by interacting with the TLR4 signaling pathway. This study rationalizes the traditional use of extracts from *O. spinosa* for therapy of urinary tract infections, due to its potential anti-inflammatory effects that are mediated *via* TLR4 receptor antagonism.

## Introduction

The roots from *Ononis spinosa* L. (Fabaceae), also called restharrow roots, are traditionally used for irrigation of the urinary tract, especially in cases of inflammation and renal gravel, and for adjuvant treatment of bacterial infections of the urinary tract (European Scientific Cooperative on Phytotherapy ESCOP, 2015). The European Medical Agency (EMA) recommends the use of restharrow extracts for flushing of the urinary tract as an adjuvant herbal material during minor urinary complaints due to its diuretic effects (EMA). Phytochemically, *O. spinosa* roots are characterized by the presence of the isoflavones trifolirhizin (Fujise et al., 1965), formononetin together with its 7-*O*-β-d-glucoside-6″-malonate (Köster et al., 1983) and 7-*O*-β-d-glucoside (syn. ononin) (Köster et al., 1983), biochanin-A-7-*O*-β-d-glucoside (Köster et al., 1983) and the pterocarpan medicarpin (Dannhardt et al., 1992); in addition, triterpenes have been described for restharrow roots, with α-onocerin as main compound (Rowan and Dean, 1972). Recently, onogenin, sativanone, calycosin (Gampe et al., 2016), and the norneolignan clitorienolactone B (Addotey et al., 2018) have been additionally identified from a restharrow root extract. Furthermore, the presence of phytosterols, especially β-sitosterol (Rowan and Dean, 1972; Haznagy et al., 1978), deoxybenzoines, especially ononetin, phenolic acids (Luczak and Swiatek, 1991), minerals, and of volatile oil (0.02%) with *trans*-anethole, carvone, menthol as main compounds has been reported (Hilp et al., 1975).

Regarding the pharmacological activity, restharrow extracts exert moderate diuretic effects, as shown within in *in vivo* rat experiments (Rebuelta et al., 1981). However, until now, it remains unclear if this is caused by the amount of essential oil in the extracts, by flavonoid glycosides or by the content of potassium salts (Hilp, 1976; Rebuelta et al., 1981). A further explanation for the diuretic activity of restharrow extracts is based on the inhibition of human hyaluronidase-1 (Hyal-1) by the isoflavone sativanone as renal Hyal-1 contributes at least in part to the control of renal fluid regulation in the kidney cells (Stridh, 2013).

As the diuretic activity of restharrow extracts seems to be only moderate, its traditional use against UTI might also be based on an anti-inflammatory potential of the herbal material. A methanolic extract from *O. spinosa* roots significantly reduced edema formation after intraperitoneal application within the Carrageenan-induced rat paw edema assay (Bolle et al., 1993). In another study, a methanolic root extract and particularly the pterocarpan medicarpin have been reported as inhibitors of the 5-lipoxygenase and leukotriene B4 formation (Dannhardt et al., 1992). Additionally, moderate inhibition of human Hyal-1, an enzyme strongly related to the induction of inflammatory cellular response, by an aqueous extract has been shown (Addotey et al., 2018); even stronger inhibition of Hyal-1 has been observed using a dichloromethane extract (Addotey et al., 2018). The inhibitory activity was related to the presence of sativanone (Addotey et al., 2018), an isoflavanone also found in decoctions (Addotey et al., 2018).

Therefore, the current study aimed at investigating the influence of a lipophilic extract as well as isolated compounds from restharrow roots on the inflammatory response as determined by interleukin 8 (IL-8) and tumor necrosis factor alpha (TNF-α) release from lipopolysaccharide (LPS)-stimulated human neutrophils. An additional aim was to investigate the potential mechanism of the observed anti-inflammatory activities.

## Materials and Methods

### General Experimental Procedures and Materials

If not stated otherwise, all chemicals were purchased from VWR (Darmstadt, Germany). Roots from *Ononis spinosa* L. were obtained from Caesar and Loretz (Hilden, Germany); batch number: 17235201, macroscopic identification was performed by JA and AH. A voucher specimen, IPBP 15332802, is deposited at the archives of the Institute of Pharmaceutical Biology and Phytochemistry, University of Münster, Germany.

### Preparation of Extracts From *O. spinosa* Roots

*Dichloromethane extract (OS1):* 1 kg of powdered restharrow root material was extracted in a Soxhlet apparatus for 8 h with 4 L of dichloromethane. The extract obtained was filtered through sea sand and anhydrous sodium sulphate, followed by evaporation of the solvent under *vacuo*. The residue was suspended in water and the mixture was lyophilized (yield 0.97% *m*/*m*, related to the starting material).

*Aqueous extract (OS2):* The extract was prepared as described by (Deipenbrock et al., 2020). Briefly, 80 g of powdered dried plant material were extracted 3 × with 800 ml water at 70°C under stirring for 2 h each. The suspension was filtrated and centrifuged for 10 min at 2600 × g. Subsequently, the extract was concentrated in *vacuo* and lyophilized (yield 15.6% *m*/*m*, related to the starting material; drug extract ratio = 6.5:1).

### Characterization of *O. spinosa* Extract OS1 (LC-MS)

For the preparation of LC-MS samples, OS1 was dissolved in a mixture of dichloromethane and methanol (1:9) to a concentration of 10 mg/ml. Chromatographic separation was performed on a Dionex Ultimate 3000 RS Liquid Chromatography System over a Dionex Acclaim RSLC 120, C18 column (2.1 × 100 mm, 2.2 µm) with a binary gradient (A: water with 0.1% formic acid; B: acetonitrile with 0.1% formic acid) at 0.4 ml/min. 0 to 9 min: linear from 5 to 100% B; 9 to 15 min: isocratic at 100% B; 15.0 to 15.1 min: linear from 100 to 5% B; 15.1 to 20 min: isocratic at 5% B for equilibration. The injection volume was 2 µl. Eluted compounds were detected using a Dionex Ultimate DAD-3000 RS over a wavelength range of 200–400 nm and a Bruker Daltonics micrOTOF-QII time-of-flight mass spectrometer equipped with an Apollo electrospray ionization source in positive mode at 3 Hz over a mass range of *m/z* 50–1,500 using the following instrument settings: nebulizer gas nitrogen, 4 bar; dry gas nitrogen, 9 L/min, 200°C; capillary voltage −4,500 V; end plate offset −500 V; transfer time 100 µs, prepulse storage 6 µs, collision energy 8 eV. MS/MS scans were triggered by AutoMS2 settings within a range of *m/z* 200–1,500, using a collision energy of 40 eV and collision cell RF of 130 Vpp. Internal dataset calibration (HPC mode) was performed for each analysis using the mass spectrum of a 10 mM solution of sodium formate in 50% isopropanol that was infused during LC re-equilibration using a divert valve equipped with a 20 µl sample loop.

Using mainly the online tool Reaxys^®^, the obtained results were finally matched with the literature regarding their sum formula, fragmentation pattern, and occurrence in the genus *Ononis*. A full MS table has been added as **Data Sheet 1**.

### Quantification of α-Onocerin in OS1

α-Onocerin was quantified by GC-FID as described by Daruházi et al. (2008). Briefly, the analysis was carried out using an Agilent 6890 instrument with a SE-30 column (0.32 mm ID × 30 m × 0.25 µm; SATO Analytik, Mönchengladbach, Germany). The temperature of the flame ionization detector was 350°C. Further settings were the same as reported by Daruházi et al. (2008). Identification of α-onocerin was performed by spiking a sample of OS1 with the isolated compound prior to the quantification experiments. Octadecane (0.15 mg/ml) was used as internal standard and quantification was performed by external calibration with the pure compound.

Blank internal standard solution was injected to confirm purity of the solvent and a blank extract solution without internal standard was analyzed to exclude that peaks were eluted at the same retention time as octadecane.

### Neutrophil Isolation, Cytokine Release, Expression of Adhesion Molecules and Cell Viability

Peripheral venous blood was taken from healthy human donors (18–35 years old) in the Warsaw Blood Donation Centre. Donors did not smoke or take any medications. They were clinically recognized to be healthy and a routine laboratory tests showed all values to be within the normal ranges. The study conformed to the principles of the Declaration of Helsinki. Each experiment was performed using cells from three different donors and assays were carried out in at least two technical replicates. Neutrophils were isolated by dextran sedimentation and centrifugation in a Ficoll Hypaque gradient (Böyum, 1968) and then re-suspended in RPMI 1640 medium supplemented with 10% Fetal Bovine Serum (FBS), 10 mM HEPES, 2 mM L-glutamine and Penicillin/Streptomycin. Extracts and compounds were dissolved in dimethyl sulfoxide (DMSO; 10 mg/ml or 10 mM stock solutions) and then diluted with RPMI 1640 medium. The extracts were tested in a concentration range of 25–100 μg/ml. Compounds were tested at concentrations of 10–100 μM. Quercetin and dexamethasone at 50 μM (0.015 and 0.02 µg/ml, resp.) were used as a positive control. The concentration of DMSO used (<0.1%) did not influence the assays. For determination of cytokine production, neutrophils were cultured in 24-well plates in the presence or absence of LPS (0.1 µg/ml) and tested extracts/compounds for 24 h at 37°C with 5% CO_2_. After 24 h, supernatants were collected and centrifuged (2,000 rpm; 10 min; 4°C). The amount of released cytokines was measured by ELISA following the manufacturer's instructions (BD Biosciences, USA). The effects on IL-8 and TNF-α production were calculated by comparing the percentages of the released agent to the control cells, which were stimulated but were not exposed to the test compounds.

For determining the surface expression of CD11b/CD18 and CD62L adhesion molecules isolated cell suspensions (1 × 10^6^) were preincubated with 100 μl of extracts/compounds for 30 min at 37°C, and LPS (1 μg/ml) was then added to the cells. After stimulation, the cells were marked with a monoclonal antibody against CD11b conjugated with phycoerythrin, PE and CD62L conjugated with fluorescein isothiocyanate (FITC; eBioscience, San Diego, CA) and analyzed by flow cytometry. The effect on the surface expression of adhesion molecules was evaluated based on a software-generated marker histogram M1 for LPS-stimulated cells.

### HEK293 Cell Culture, IL-8 Release, and Cell Viability

In general, experiments were performed as described in detail by Saenger et al. (2019). Briefly, TLR4-transfected human embryonic kidney (HEK293) cells (TLR4^+^ cells) and non-transfected control cells (TLR4^−^ cells) were cultured according to the manufacturer's protocols (InvivoGen, San Diego, USA). Prior to the assay, 30,000 cells were seeded out in 200-µl medium per well in a 96-well microtiter plate. After 24 h of cultivation (37°C, 5% CO_2_) the medium was removed from each well and 200 µl of medium containing the respective test solution were added. OS1 and OS2 were tested at 1, 10 and 100 µg/ml each, 1% DMSO was used as a solubilizer and was therefore included also into the respective negative control. LPS-free controls, containing test solution only, were additionally included. After 1 h of incubation, 50 µl of medium, supplemented with LPS (16 ng/ml final concentration, Sigma-Aldrich, München, Germany) were added. In an additional experiment, cells were incubated with OS1 and OS2 at 100 µg/ml as described, but after 1 h of incubation, the extract solutions were removed and 250-µl LPS (16 ng/ml) were added. For the co-incubation experiment, each extract was incubated with LPS at 37°C. After 1 h, 50 µl of the respective sample were added to each well, containing 200 µl of cell culture medium (final concentrations as described above).

After 3 h of stimulation with LPS, 150 µl of the supernatant were transferred to a new 96-well microtiter plate and its IL-8 concentration was determined using a Human IL-8/CXCL8 DuoSet ELISA Kit (R&D Systems, Minneapolis, USA) following the manufacturer's instructions. MaxiSorp 96-well microtiter plates were used for antibody immobilization. Incubations were performed at 25°C at 600 rpm in a microtiter plate shaker. Finally, the absorption was measured at λ = 450 nm (reference wavelength λ = 570 nm) in a microplate photometer (Tecan Sunrise, Tecan GmbH, Griesheim).

In addition, the cell viability was assessed by MTT assay (Mosmann, 1983). For that, the cell culture medium was removed completely after transferring the supernatant and cells were washed with 200 µl of phosphate buffered saline (PBS). Subsequently, PBS was substituted by 50 µl of MTT-reagent (5 mg/ml in medium) and cells were incubated for 4 h (37°C, 5% CO_2_). The reagent was removed, crystals were dissolved in 100-µl DMSO and the absorption of the solution was measured at λ = 570 nm (reference wavelength λ = 630 nm).

### Statistical Analysis

Statistical analyses were performed using Statistica 13.1 software. GraphPad Prism version 3.00 or 5.01 for Windows (GraphPad Software, La Jolla California USA) was used to plot the data. The results were expressed as mean ± SEM from at least three independent experiments. The statistical significance of the differences in means was established by ANOVA with Dunnett's or Tukey's *post hoc* test. *P*-values below 0.05 were considered statistically significant.

## Results

**Figure 1:**
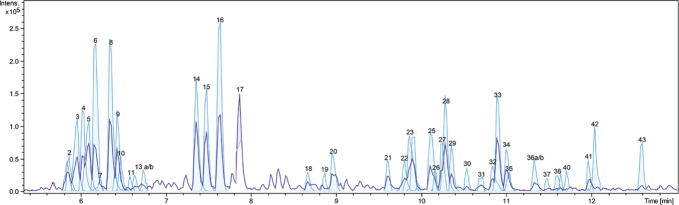
Detail of base-peak chromatogram of OS1. Peaks are numbered and identified as listed in **Table 1**.

A dichloromethane extract (OS1) was prepared from the roots of *O. spinosa* by Soxhlet extraction. OS1 was obtained in a yield of 0.97% (*m*/*m*), related to the starting material and characterized using LC-qTOF-MS. **Figure 1** shows the respective chromatogram. Peaks were assigned to the respective secondary metabolites as displayed in **Table 1**, by comparison of adduct ions and major fragments' exact *m/z* values with published data of *Ononis* constituents.

**Table 1:** LC-qTOF-MS peak characteristics as obtained by integrating a Full-MS-chromatogram of *O. spinosa* root extract OS1.

Cmpd. no.	*t*R [min]	Peak Area	*m/z* [M+H] ^+^	MS/MS fragments	Ion formula [M+H]^+^	Error [mDa]	mSigma	Compound	Reference
1	5.851	27249	602.2244	84.0822, 135.0442, 144.1006, 163.0397, 287.0922, 288.1383, 315.0864	C_30_H_36_NO_12_	-1.2	6.8	Onogenin 7-*O*-*β*-d-glucoside 6''-piperidine 2-acetate	(Gampe et al., 2019)
2	5.875	28070	431.1337	269.0813	C_22_H_23_O_9_	0.1	2.8	Formononetin 7-*O*-*β*-d-glucoside (Ononin)	(Gampe et al., 2019)
3	5.968	23006	588.2442	84.0837, 135.0437, 144.1011, 163.0451, 273.1112, 288.1465, 301.1072	C_30_H_38_NO_11_	-1.6	9.5	Sativanone 7-*O*-*β*-d-glucoside 6''-piperidine 2-acetate	(Gampe et al., 2019)
4	6.039	57116	477.1393	135.0435, 163.0389, 177.0550, 229.0847, 257.0824, 287.0907, 315.0872	C_23_H_25_O_11_	0.2	4.8	Onogenin 7-*O*-*β*-d-glucoside	(Gampe et al., 2019)
5	6.101	71064	558.2338	84.0819, 137.0594, 144.1015, 161.0597, 271.0973, 288.1437	C_29_H_36_NO_10_	0.5	3.1	Medicarpin 3-*O*-*β*-d-glucoside 6''-piperidine 2-acetate	(Gampe et al., 2019)
6	6.184	49004	463.1600	123.0427, 135.0441, 151.0459, 163.0416, 273.1120	C_23_H_27_O_10_	-0.1	83.6	Sativanone 7-*O*-*β*-d-glucoside	(Gampe et al., 2019)
7	6.241	1049	447.1283	123.0438, 151.0380, 175.0372, 255.0608, 285.0749	C_22_H_23_O_10_	-0.3	38.5	Maackiain 3-*O*-*β*-d-glucoside	(Gampe et al., 2019)
8	6.362	17770	433.1484	79.0560, 109.0659, 137.0589, 161.0617, 271.0964	C_22_H_25_O_9_	-0.9	5.7	Medicarpin 3-*O*-*β*-d-glucoside	(Gampe et al., 2019)
9	6.442	63964	313.1069	107.0491, 151.0396, 207.0783, 223.0750, 253.0879	C_18_H_17_O_5_	0.2	2.7	Clitorienolactone B	(Addotey et al., 2018; Gampe et al., 2019)
10	6.481	17946	289.1058	99.0460, 118.0416, 146.0346, 161.0584, 187.0732	C_16_H_17_O_5_	-1.3	14.2	11b-Hydroxy-11b,1-dihydromedicarpin	(Barrero et al., 1994)
11	6.589	1514	519.1506	123.0411, 137.0595, 161.0550, 271.0978	C_25_H_27_O_12_	-0.9	8.2	Medicarpin 3‐*O*‐*β*‐d‐glucoside-6″‐malonate	(Gampe et al., 2019)
12	6.645	7859	473.1444	269.0807	C_24_H_25_O_10_	-0.2	24.2	6''-*O*-Acetylononin	(Barrero et al., 1989)
13a	6.751	4758	285.0759	107.0491, 123.0425, 137.0499, 151.0390, 198.0599, 213.0568	C_16_H_13_O_5_	0.1	75.9	Maackiain	(Gampe et al., 2019)
13b	6.751	7948	519.1485	135.0432, 163.0383, 177.0538, 287.0900, 315.0853	C_25_H_27_O_12_	1.2	17.1	Onogenin acetylhexoside	
14	7.366	107816	269.0808	118.0404, 137.0244, 170.0712, 181.0652, 197.0601, 213.0886, 226.0609, 237.0538, 253.0494	C_16_H_13_O_4_	-0.7	73.6	Formononetin	(Gampe et al., 2019)
15	7.482	91797	315.0850	107.0493, 135.0435, 147.0439, 163.0374, 197.0588, 229.0857	C_17_H_15_O_6_	1.3	5.7	Onogenin	(Gampe et al., 2019)
16	7.648	122655	301.1067	107.0488, 121.0637, 135.0450, 147.0435, 163.0388, 213.0906, 267.0667	C_17_H_17_O_5_	-0.3	7.1	Sativanone	(Addotey et al., 2018; Gampe et al., 2019)
17	7.871	88569	271.0959	123.0438, 137.0588, 161.0587	C_16_H_15_O_4_	-0.6	4.6	Medicarpin	(Gampe et al., 2019)
18	8.672	14117	283.0947	121.0606, 150.0295, 169.0668, 197.0579, 211.0736, 240.0772, 267.0626	C_17_H_15_O_4_	-1.8	11.5	Anhydrovariabilin	(Barrero et al., 1994)
19	8.866	2233	455.3456	277.2127, 295.2250, 335.2175,	C_30_H_47_O_3_	6.4	26.8	putative triterpenoid	
20	8.966	25981	473.3604	95.0832, 109.1002, 119,0862, 123.1139, 189.1623, 235.1673	C_30_H_49_O_4_	2.1	8.9	putative triterpenoid	
21	9.610	20364	471.3442	109.0994, 119.0849, 135.1103, 219.1735, 233.1556, 435.3171, 453.3267	C_30_H_47_O_4_	2.7	8.1	putative triterpenoid	
22	9.801	9654	471.3437	107.0860, 119.0870, 133.0993, 147.1160, 175.1145, 193.1197, 217.1583, 453.3309	C_30_H_47_O_4_	3.1	12	putative triterpenoid	
23	9.862	25310	279.2298	81.0698, 95.0843, 109.1013, 131.0844	C_18_H_31_O_2_	2	3.1	putative fatty acid derivative	
24	9.909	26645	279.2298	81.0699, 95.0846, 105.0684. 109.1013. 131.0841	C_18_H_31_O_2_	-2	16.5	putative fatty acid derivative	
25	10.119	27712	277.2146	81.0694, 91.0541, 105.0663, 119.0823, 131.0861, 163.1070	C_18_H_29_O_2_	-1.6	34.9	putative fatty acid derivative	
26	10.172	2332	295.2249	79.0537, 93.0693, 105.0690, 119.0863, 151.1105, 163.1139	C_18_H_31_O_3_	1.8	26.9	putative fatty acid derivative	
27	10.240	25773	277.2148	79.0481, 91.0535, 105.0694, 199.0879, 133.0243, 161.1238	C_18_H_29_O_2_	-1.4	12.3	putative fatty acid derivative	
28	10.286	71904	457.3651	95.0829, 109.1005, 121.0995, 135.1151, 147.1151, 161.1253, 177.1635. 189.1619, 231.1741. 303.2285	C_30_H_49_O_3_	2.5	2.1	putative triterpenoid	
29	10.357	20859	277.2148	81.0320, 93.0642, 109.0597, 119.0799	C_18_H_29_O_2_	1.4	13.2	putative fatty acid derivative	
30	10.534	5947	455.3487	95.0874, 107.0830, 121.1001, 135.1139, 149.0983, 161.1290, 175.1480, 189.1575, 247.1727, 301.2133	C_30_H_47_O_3_	-3.3	125.5	putative triterpenoid	
31	10.701	3652	439.3540	107.0849, 109.0998, 119.0844, 131.0853, 147.1153, 161.1315. 175.1440, 187.1481, 227.0654	C_30_H_47_O_2_	-3.1	45	putative triterpenoid	
32	10.836	18738	457.3645	95.0840, 107.0862, 109.0997, 121.1010, 133.1017, 147.1151, 161.1320, 175.1439, 201.1629, 215.1779	C_30_H_49_O_3_	3.1	15.7	putative triterpenoid	
33	10.895	81439	455.3500	95.0858, 109.1000, 119.0855, 121.0990, 133.0991, 147.1143, 161.1270, 175.1411, 189.1628, 203.1754, 303.2301	C_30_H_47_O_3_	-2	29.8	putative triterpenoid	
34	11.998	33172	441.3699	95.0847, 109.1002, 119.0866, 121.1015, 133.1004, 135.1139, 147.1157, 161.1315, 163.1467, 175.1463, 189.1609, 203.1797	C_30_H_49_O_2_	-2.8	8	putative triterpenoid	
35	11.023	9969	453.3338	95.0829, 107.0877, 121.0974, 133.1000, 149.0978, 163.1134, 175.1434, 301.2160	C_30_H_45_O_3_	2.5	33.8	putative triterpenoid	
36a	11.330	4249	439.3555	95.0857, 107.0834, 119.0839, 121.1026, 161.1306, 173.1290	C_30_H_47_O_2_	1.5	30.4	putative triterpenoid	
36b	11.330	11981	445.3652	97.0650, 109.0656, 133.0930, 145.0978, 271.1997	C_29_H_49_O_3_	2.5	41	putative triterpenoid	
37	11.471	2629	279.2289	79.0547, 81.0672, 95.0824, 109.1031, 123.1136, 161.5783, 179.3368	C_18_H_31_O_2_	-2.9	23.6	putative fatty acid derivative	
38	11.591	3279	485.3597	95.0837, 109.1054, 121.0994, 135.116, 147.1160, 163.1342, 189.1549, 203.1700, 249.1840, 303.2281	C_31_H_49_O_4_	-2.8	-5.9	putative triterpenoid	
39	11.635	1104	295.2247	91.0490, 121.0992, 149.0514	C_18_H_31_O_3_	2.1	92.2	putative fatty acid derivative	
40	11.703	4007	483.3483	89.0628, 121.1001, 133.0877, 175.1400, 189.1555, 285.2188, 331.2329	C_31_H_47_O_4_	1.4	33.9	putative triterpenoid	
41	11.959	15470	423.3598	95.0848, 107.0854, 121.1001, 133.1002, 135.1164, 147.1164, 163.1474, 173.1324, 213.1607, 269.2255	C_30_H_47_O	2.3	12.5	putative triterpenoid	
42	12.033	5378	423.3582	95.0848, 107.0854, 119.0843, 121.1001, 133.1002, 135.1164, 147.1164, 163.1474, 173.1324, 213.1607, 269.2255	C_30_H_47_O	3.9	20.7	putative triterpenoid	
43	12.582	4940	439.3555	95.0874, 107.0864, 109.0983, 119.0853, 121.1009, 133.0993, 135.1116, 147.1146, 159.1125, 175.1430, 203.1767	C_30_H_47_O_2_	1.6	115.2	putative triterpenoid	

Peaks were assigned to known constituents of the genus *Ononis* as denominated in the references. Assignments without references are based on the *de novo* interpretation of MS, MS², and UV-spectra.

The same analytical procedure was performed to characterize the aqueous extract OS2; results have been reported recently (Deipenbrock et al., 2020). OS1 and OS2 contained high amounts of flavonoids, especially isoflavones and pterocarpans (OS1) or their respective glycosides (OS2) as representative compounds for the genus *Ononis*. Unfortunately, much fewer flavonoid aglyca have previously been described for *Ononis* species compared to flavonoid glycosides, particularly with respect to their fragmentation pattern. Therefore, the number of identified flavonoids given in **Table 1** is comparably short, but includes all major peaks detected in the chromatogram. In addition, several saponins were detected in OS2, whereas OS1 contained the respective triterpenes and triterpene acid aglyca. α-Onocerin as one of the most abundant compounds reported for *O. spinosa*, was not detected in the LC-MS screening due to the lack of a chromophor as well as its apparently poor ability to ionize. Therefore, quantification by GC-FID was performed in addition to the LC-MS characterization, confirming the presence of the compound in the extract. A content of 10.5% (*m*/*m*) related to the crude extract supported α-onocerin as one major constituent.

**Figure 2:**
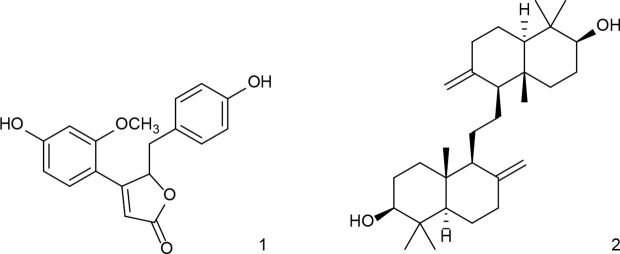
Structures of clitorienolactone B (**1**) and α-onocerin (**2**) isolated from a dichloromethane extract of the roots of *O. spinosa*.

**Figure 3:**
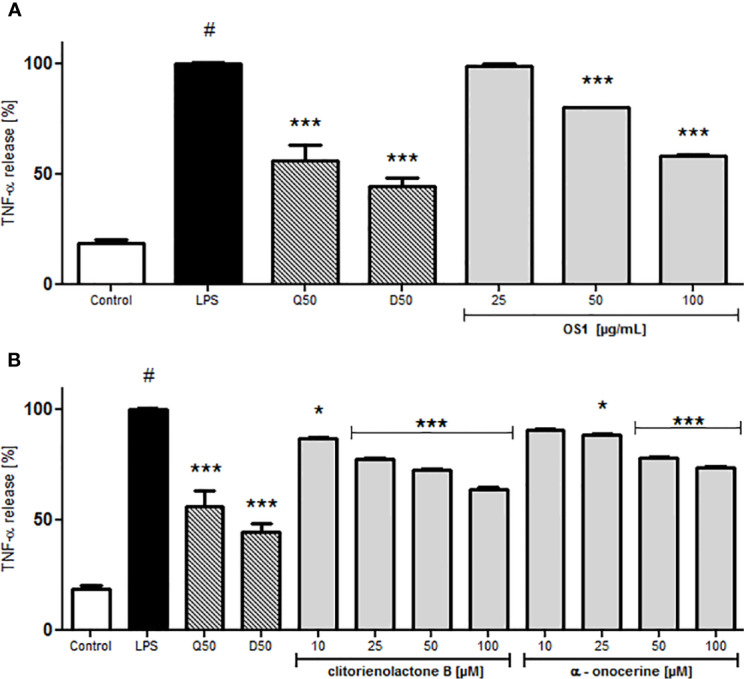
Effect of *O. spinosa* L. extract OS1 **(A)** and isolated compounds clitorienolactone B and α-onocerin **(B)** at concentrations of 25 to 100 μg/ml and 10 to 100 μM, respectively, on TNFα release from LPS-stimulated human neutrophils. Positive controls: quercetin (Q) and dexamethasone (D), each 50 μM. Data are expressed as mean ± SEM, originating from 3 independent experiments performed on neutrophils, isolated from independent donors; each experiment has been performed with n = 3 technical replicates. Statistical significance: *p < 0.05, ***p < 0.001 versus stimulated control (Dunnett's *post hoc* test). # statistically significant (*p* < 0.001) versus non-stimulated control; LPS, LPS stimulated control; Control, non-stimulated control.

**Figure 4:**
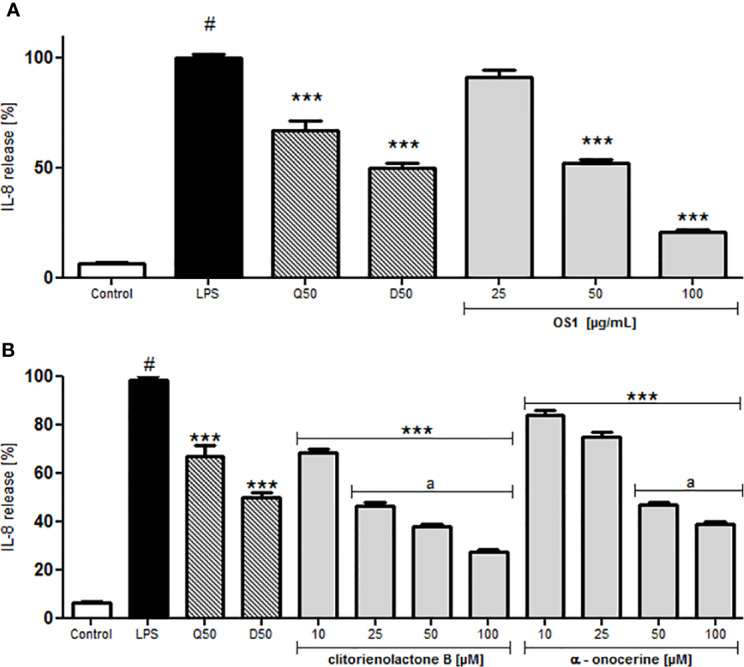
Effect of *O. spinosa* L. extract OS1 **(A)** and isolated compounds clitorienolactone B and α-onocerin **(B)** at concentrations of 25 to 100 μg/ml and 10 to 100 μM, respectively, on IL-8 release from LPS-stimulated human neutrophils. Positive controls: quercetin (Q), dexamethasone (D) 50 μM. Data are expressed as mean ± SEM from three independent experiments performed on neutrophils isolated from independent donors, n = 3 technical replicates. Statistical significance: ***p < 0.001 versus stimulated control (Dunnett's *post hoc* test), #: statistically significant (*p* < 0.001) versus non-stimulated control; LPS, LPS stimulated control; Control, non-stimulated control; a, statistically significant (*p* < 0.001) versus quercetin.

For investigations of potential anti-inflammatory activity, human primary neutrophils were incubated with LPS for 24 h with or without addition of OS1. Further, the rare norneolignan clitorienolactone B (**1**) and α-onocerin (**2**) (**Figure 2**), two compounds previously isolated from OS1 (Addotey et al., 2018) were included in the assay as they both represent major compounds within the extract Despite its high abundance, only few studies have dealt with bioactivities of α-onocerin. Subsequently, quantification of released IL-8 and TNF-α was performed by ELISA. Dexamethasone and quercetin (50 μM) served as positive controls, leading to a significantly reduced cytokine secretion (**Figures 3** and **4**). OS1 at 50 and 100 μg/ml significantly supressed the LPS-induced TNF-α secretion in a concentration-dependent manner (**Figure 3A**). This effect was possibly correlated to the norneolignan clitorienolactone B and the triterpene α-onocerin, which both showed significant inhibition of LPS-induced effects at 10 and 25 μM resp. (**Figure 3B**). Similar results were obtained for IL-8 formation: OS1 strongly reduced LPS-induced IL-8 secretion at ≥50 µg/ml (**Figure 4A**), again based on the inhibitory effects of clitorienolactone B and α-onocerin which were active at concentrations ≥10 µM (**Figure 4B**).

For a better understanding of the anti-inflammatory activity of OS1 and the isolated compounds, their respective influence on selected cell adhesion molecules was also investigated. Testing was performed on the expression of the cell surface receptor Macrophage-1 antigen (Mac-1) consisting of CD11b (integrin α-M) and CD18 (integrin β-2). Integrins are heterodimers, characterized by two, non-covalently linked proteins, designated as either α- or β-protein. Leukocyte integrins are a subfamily with the same β2 chain (syn. CD18), but interacting with different α chains (e.g., αL, αM, and αX). Mac1 (CD11b/CD18, α_M_β_2_) expression on the cell membrane is stimulated during inflammatory response and therefore Mac-1 is regarded as key player for phagocytosis, transendothelial phagocyte migration, and activation of macrophages or neutrophils (Tan et al., 2000). It can be activated in polymorphonuclear leucocytes (PMNs) *via* activation of TLR4 (Zhou et al., 2005).

**Figure 5:**
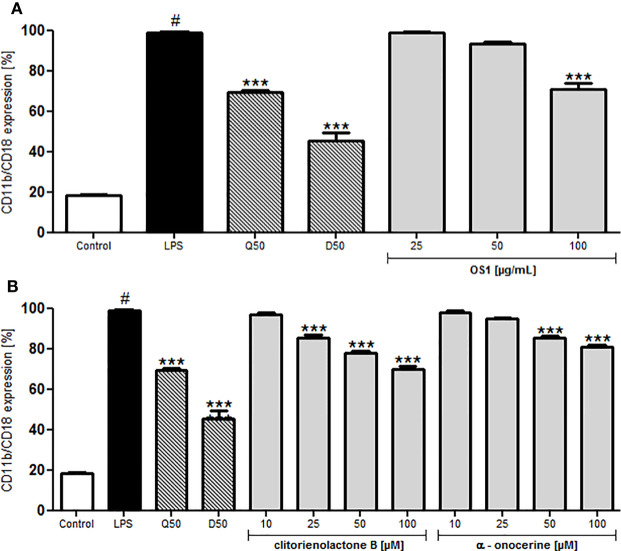
Effect of *O. spinosa* root extract OS1 **(A)**, clitorienolactone B and α-onocerin **(B)** at concentrations of 25 to 100 μg/ml and 10 to 100 μM, respectively, on CD11b/CD18 expression by LPS-stimulated neutrophils. Positive controls: quercetin (Q), dexamethasone (D), 50 μM. Data are expressed as mean ± SEM from independent experiments performed on human neutrophils, isolated from independent donors; n = 3 technical replicates. Statistical significance: ***p < 0.001 versus stimulated control (Dunnett's *post hoc* test), #: statistically significant (*p* < 0.001) versus non-stimulated control; LPS, LPS stimulated control; Control, non-stimulated control.

LPS significantly induced the Mac-1 (CD11b/CD18) expression of human neutrophils (**Figure 5**). OS1 at the highest tested concentration of 100 μg/ml inhibited Mac-1 expression significantly by about 30% (**Figure 5A**) and also clitorienolactone B and α-onocerin showed concentration dependent inhibition of this surface marker (**Figure 5B**).

**Figure 6:**
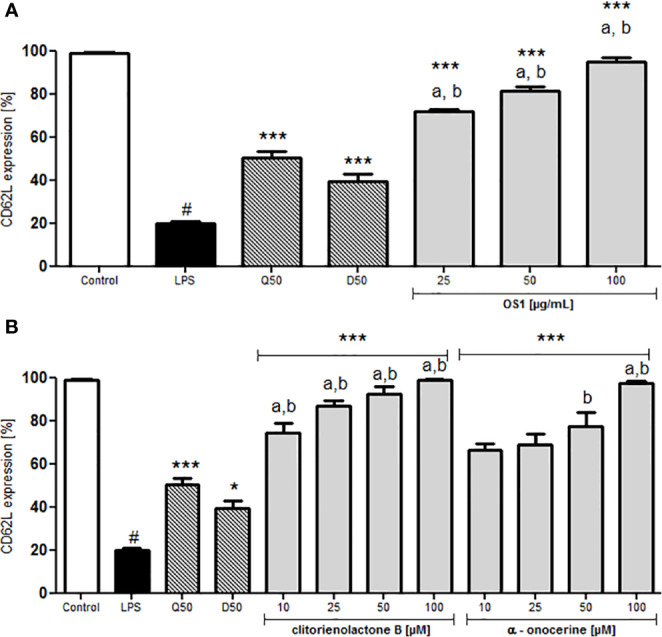
Effect of *O. spinosa* root extract OS1 **(A)**, clitorienolactone B and α-onocerin **(B)** at concentrations of 25 to 100 μg/ml and 10 to 100 μM, respectively, on CD62L expression by LPS-stimulated neutrophils. Positive controls: quercetin (Q), dexamethasone (D) 50 μM. Data are expressed as mean ± SEM from independent experiments performed on human neutrophils, isolated from independent donors; n = 3 technical replicates. Statistical significance: *p < 0.05, ***p < 0.001 versus stimulated control (Dunnett's *post hoc* test); # statistically significant (*p* < 0.001) versus non-stimulated control; LPS, LPS stimulated control; Control, non-stimulated control; a, statistically significant (*p* < 0.001) versus quercetin; b, statistically significant (*p* < 0.001) versus dexamethasone.

In case of inflammation, specific trafficking of lymphocytes *via* the blood stream and crossing the endothelial barrier toward the inflamed tissue or lymphoid tissue is desirable. Typically, T-cells express L-selectin (CD62L), interacting with the glycosylation-dependent cell adhesion molecule-1 (GlyCAM-1) on the endothelial barrier (Wedepohl et al., 2012). CD62L can act as a low-affinity receptor for LPS (Malhotra and Bird, 1997). To investigate the effect of OS1 on CD62L expression, human neutrophils were treated with LPS, leading to a significantly reduced L-selectin expression (**Figure 6**). Dexamethasone and quercetin-treated control groups showed higher CD62L expression compared to the LPS-stimulated control (**Figure 6**). Pre-treatment of the cells with OS1 resulted in concentration-dependent and significantly higher L-selectin expression than in the respective dexamethasone and quercetin-treated groups (**Figure 6A**). In addition, clitorienolactone B had a significant influence on L-selectin expression (**Figure 6B**). Lower activity was detected for α-onocerin treated cells (**Figure 6B**).

Up-regulation of Mac-1 (CD11b/CD18), the reduced expression of L-selectin and secretion of IL-8 can be mediated by the signaling pathway *via* Toll-like receptors (TLR) 2 or 4 (Sabroe et al., 2003), as well as the release of TNF-α (Lee and Kim, 2007; Aomatsu et al., 2013).

In order to assess whether TLR4 was involved in the anti-inflammatory effects observed in the neutrophils, commercially available human embryonic kidney cells (HEK293), transfected with TLR4 and co-factors MD2 (myeloid differentiation factor 2)/CD14 (TLR4^+^ cells) were used. TLR4 recognizes a multitude of molecules. Hence, criteria have been postulated for the differentiation of molecules directly and indirectly activating TLR4. Conversely, also to take the possibility of an indirect inhibition into account, OS1 was tested in two scenarios. The first scenario assumed that the extract OS1 contained an inhibitor, directly interacting with TLR4. TLR4^+^ cells were therefore preincubated with OS1 prior to the addition of LPS. The second scenario assumed that certain compounds within the extract exerted an indirect inhibitory effect due to binding to LPS. Thus, in a second experiment, instead of preincubating the TLR4^+^ cells with OS1, the extracts were co-incubated with LPS for 1 h prior to addition of this mixture to the cells.

Subsequently, the IL-8 secretion was quantified by ELISA. Since aqueous preparations, rather than dichloromethane extracts are mainly used in traditional medicine, an aqueous extract (OS2) was also included in all assays.

**Figure 7:**
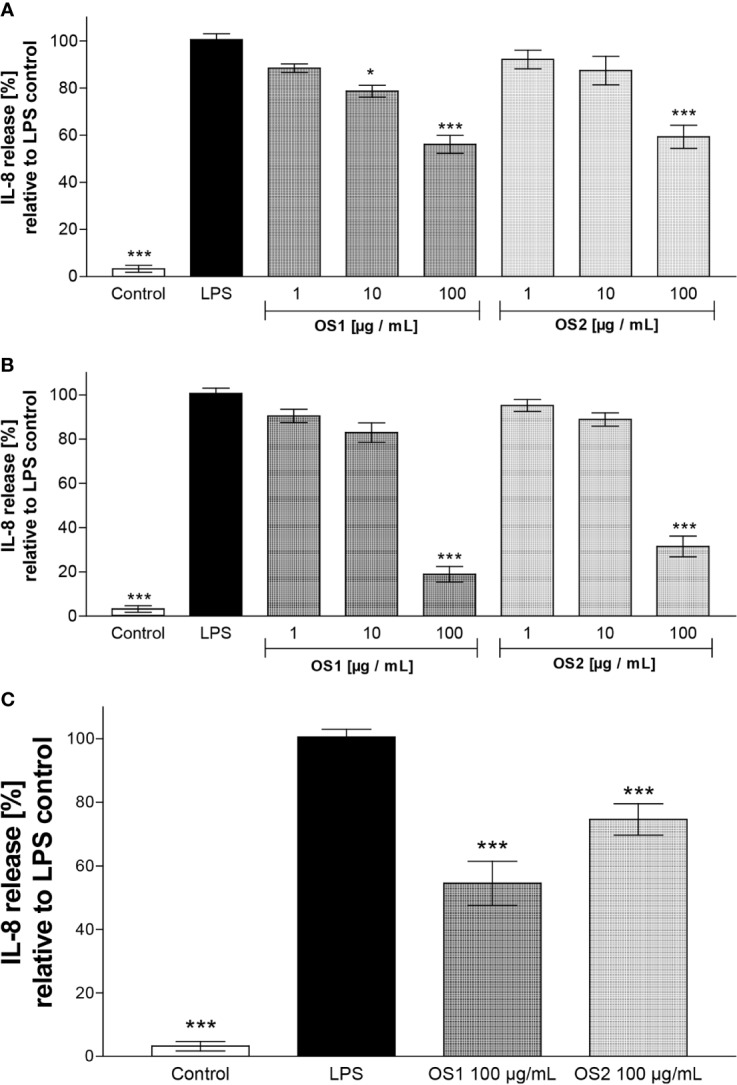
**(A)** Effect of *O. spinosa* root extracts OS1 and OS2 at concentrations of 1 to 100 µg/ml on the IL-8 release from LPS-stimulated TLR4^+^ transfected HEK293 cells. **(B)** IL-8 secretion after preincubation of OS1 and OS2 with LPS resp. prior to addition of the mixture to the cells. **(C)** IL-8 secretion from TLR4+ cells pretreated with OS1 and OS2 at 100 µg/ml for 1 h before addition of LPS. Data are expressed as mean ± SEM from three independent experiments with n = 4 technical replicates. *p < 0.05, ***p < 0.001 (one-way ANOVA, Tukey's post-test), compared to LPS-stimulated control (LPS); control, non-stimulated control.

As shown in **Figure 7A**, both extracts showed a significant inhibition of the LPS-induced IL-8 release at 100 µg/ml. With a reduction to 56% compared to the LPS control, OS1 exerted a slightly stronger inhibition than OS2 (59% IL-8 secretion relative to LPS control). The IL-8 secretion for OS1 was significantly lower than the LPS-stimulated control even at 10 µg/ml (reduction to 79%). The non-transfected TLR4^-^ cells did not show any cytokine release; the cell viability was not affected by the extracts either (**Supplementary Figure 1**).

When the extracts were allowed to interact with LPS prior to addition to the cells (**Figure 7B**), a further decrease in the IL-8 release at 100 µg/ml to 19% and 31% was observed for OS1 and OS2, respectively. This result implies an indirect anti-inflammatory effect *via* interaction of certain compounds within the extracts with LPS.

In order to clarify, if there were direct effects of OS1 and OS2 at TLR4 at all, a third experiment was performed. As in the first experiments, extracts were incubated with TLR4^+^ cells, but the extract solution was completely removed before addition of LPS. As displayed in **Figure 7C**, a decrease in IL-8 secretion was still observed for OS1 (55%) and OS2 (75%), although for OS2 the reduction was weaker than observed after co-incubation with LPS (**Figure 7B**).

## Discussion

Restharrow roots are widely used for UTI and for removal of renal gravel. While older literature and textbooks emphasize on the diuretic activity of the herbal material, anti-inflammatory activities of *O. spinosa* extracts are moving more into focus. This is supported by *in vivo* animal studies, and by different molecular targets identified within different *in vitro* studies related to inflammatory cellular response. It seems interesting that different extracts and compounds from restharrow roots influence the inflammatory process in a pleiotropic manner: the methanolic extract and the pterocarpan medicarpin inhibit the 5-lipoxygenase and subsequently the leukotriene formation (Dannhardt et al., 1992). Dichloromethane extract and the non-glycosylated isoflavonoid sativanone (and also with higher IC_50_ compared to sativanone the isoflavones onogenin and calycosin-D) as well as medicarpin are inhibitors of Hyal-1, a key enzyme for inflammatory cellular response (Addotey et al., 2018). The present study additionally indicated anti-inflammatory effects of the dichloromethane extract OS1, of the norneolignan clitorienolactone B and the triterpene α-onocerin on the LPS-induced secretion of IL-8 and TNF-α in human neutrophils. This inhibition of cytokine release could be explained by antagonism of TLR4 or an interaction with proteins of the TLR4-signaling pathways.

In addition to a direct interaction with TLR4, both of the extracts seemed to reduce the secretion of IL-8 even further by interacting with LPS. Such a mechanism has been described, e.g., for the antibiotic polymyxin B (Morrison and Jacobs, 1976), which is a common standard to neutralize biological effects of LPS (for review, see Bhor et al., 2005). In addition, modifications in the Lipid A structure or conformation of LPS can reduce its ability to effectively activate the signaling pathway (for review see Rietschel et al., 1994 and Peri and Calabrese, 2014). Besides Lipid A mimetics and synthetic small molecules, also a set of natural products has been described to modulate TLR4 activity *in vitro* (Peri and Calabrese, 2014). Although these compounds were generally not structurally related and their modes of TLR4 modulation were different, interestingly, a major part of these anti-inflammatory natural products belonged to the large class of polyphenols. As part of another study, anti-inflammatory activity of the isoflavone Biochanin A has been described, however, by directly inhibiting the activation of NFκB, the major downstream transcription factor within the TLR4 signaling pathway (Kole et al., 2011). The same compound has also been found to reduce the expression of signal proteins of the TLR/NFκB pathway, such as TLR4/MyD88 and TIRAP (Wu et al., 2018), supporting an interaction of isoflavonoids with this signaling pathway.

On the other hand, also the triterpene saponin glycyrrhizin was reported to inhibit the formation of the LPS.MD-2.TLR4 complex (Peri and Calabrese, 2014). Whether therefore the isoflavonoids or the triterpenes and triterpene saponins present in OS1 or OS2 are responsible for the reduction of IL-8 secretion in TLR4^+^ cells, and in what way exactly the extracts and their active compounds interact with LPS or TLR4, remains a topic for further research.

In summary, it becomes obvious that restharrow root extracts act anti-inflammatory on multiple targets. Also taking into account the moderate diuretic effect on the kidney by an inhibition of the renal Hyal-1 by the isoflavonoids (Addotey et al., 2018), such additive pharmacodynamic effects can be of benefit for the patients within the therapy of UTI.

## Data Availability Statement

The datasets generated for this study are available on request to the corresponding author.

## Author Contributions

VS, BG, TS, and JS performed experiments and evaluated data. VS, AH, and AK wrote the MS. JA donated compounds. JA, BG, VS, TS, AH, AK, and JJ contributed conception and design of the study. AH, AK and JJ mentored the study and reviewed experimental data. All authors contributed to the article and approved the submitted version.

## Funding

This work was entirely performed without external funding, only with intramural university grants.

## Conflict of Interest

The authors declare that the research was conducted in the absence of any commercial or financial relationships that could be construed as a potential conflict of interest.

## Abbreviations

CD18, integrin β-2; CD11b, integrin α-M; CD62L, L-selectin; DMSO, dimethyl sulfoxide Hyal-1, human hyaluronidase-1; IL-8, interleukin 8; LPS, lipopolysaccharide; PBS, phosphate buffered saline; TNF-α, tumor necrosis factor alpha; TLR4, Toll-like receptor 4; UTI, urinary tract infections.
