# Supplementary material for: Root Extracts From Ononis spinosa Inhibit IL-8 Release via Interactions With Toll-Like Receptor 4 and Lipopolysaccharide
Source: Front Pharmacol. 2020 Jun 12;11:889. doi: 10.3389/fphar.2020.00889 (PMC7304261; doi:10.3389/fphar.2020.00889)

Supplementary Material

Root Extracts from Ononis spinosa inhibit IL-8 Release *via* Interactions with Toll-like receptor 4 and Lipopolysaccharide

Verena Spiegler[1,*#], Barbara Gierlikowska[2,*], Thorsten Saenger[3], John Addotey[4], Jandirk Sendker[1], Joachim Jose[3], Anna K. Kiss[5], Andreas Hensel[1]

[1] *University of Münster, Institute of Pharmaceutical Biology and Phytochemsitry, Münster, Germany*
[2] *Department of Laboratory Diagnostics and Clinical Immunology of Developmental Age, Medical University of Warsaw, Warsaw, Poland*
[3] *University of Münster, Institute of Pharmaceutical and Medicinal Chemistry, Münster, Germany*
[4] *Department of Pharmaceutical Chemistry, Faculty of Pharmacy and Pharmaceutical Sciences, Kwame Nkrumah University of Science and Technology, Kumasi, Ghana*
[5] *Department of Pharmacognosy and Molecular Basis of Phytotherapy, Medical University of Warsaw, Warsaw, Poland*

[*] Contributed equally to this publication

[#] Correspondence: Dr. Verena Spiegler

Verena.Spiegler@uni-muenster.de

Phone +49 251 8333376; fax +49 251 8338341.

Supplementary Figure 1: Viability of $TLR4^{+}$ HEK293 cells after treatment with extracts OS1 or OS2 from *O. spinosa* as determined by MTT assay.

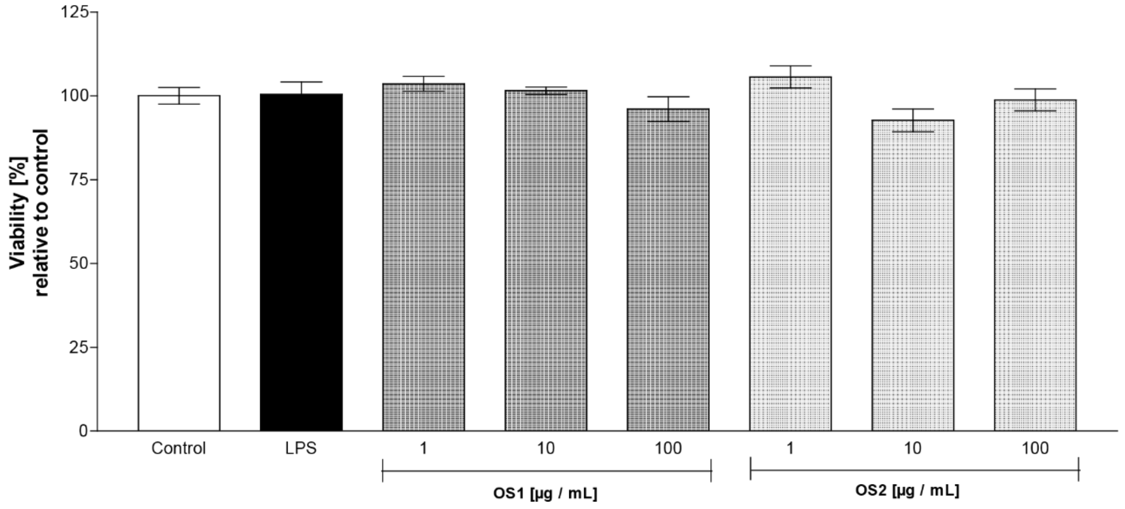

Supplement: Supplementary file 2 [file Image_1.pdf]
